# Optimization of the hydrogen response characteristics of halogen-doped SnO_2_

**DOI:** 10.1038/s41598-023-29312-6

**Published:** 2023-02-13

**Authors:** Petros-Panagis Filippatos, Rohit Sharma, Anastasia Soultati, Nikolaos Kelaidis, Christos Petaroudis, Anastasia-Antonia Alivisatou, Charalampos Drivas, Stella Kennou, Stavros-Richard G. Christopoulos, Dimitris Davazoglou, Maria Vasilopoulou, Alexander Chroneos

**Affiliations:** 1 Institute of Nanoscience and Nanotechnology (INN), National Center for Scientific Research Demokritos, Agia Paraskevi, 15341 Athens, Greece; 2 Faculty of Engineering, Environment and Computing, Coventry University, Priory Street, Coventry, CV1 5FB UK; 3 Department of Electrical and Electronics Engineering, Faculty of Engineering, University of West Attica, Campus 2, No. 250, Thivon Str., 12244 Athens, Greece; 4 School of Mining and Metallurgical Engineering, National Technical University of Athens, 9 Iroon Polytechniou Str., Zografou Campus, 15780 Athens, Greece; 5 Department of Chemical Engineering, University of Patras, 26504 Patras, Greece; 6 Department of Electrical and Computer Engineering, University of Thessaly, 38221 Volos, Greece; 7 Department of Materials, Imperial College, London, SW7 2AZ UK

**Keywords:** Chemistry, Engineering, Materials science, Physics

## Abstract

The increasing demand for efficient sensing devices with facile low-cost fabrication has attracted a lot of scientific research effort in the recent years. In particular, the scientific community aims to develop new candidate materials suitable for energy-related devices, such as sensors and photovoltaics or clean energy applications such as hydrogen production. One of the most prominent methods to improve materials functionality and performance is doping key device component(s). This paper aims to examine in detail, both from a theoretical and an experimental point of view, the effect of halogen doping on the properties of tin dioxide (SnO_2_) and provide a deeper understanding on the atomic scale mechanisms with respect to their potential applications in sensors. Density Functional Theory (DFT) calculations are used to examine the defect processes, the electronic structure and the thermodynamical properties of halogen-doped SnO_2_. Calculations show that halogen doping reduces the oxide bandgap by creating gap states which agree well with our experimental data. The crystallinity and morphology of the samples is also altered. The synergy of these effects results in a significant improvement of the gas-sensing response. This work demonstrates for the first time a complete theoretical and experimental characterization of halogen-doped SnO_2_ and investigates the possible responsible mechanisms. Our results illustrate that halogen doping is a low-cost method that significantly enhances the room temperature response of SnO_2_.

## Introduction

The advent of the hydrogen economy will be advantageous due to the comparably lower CO_2_ fingerprint and the high energy density^1,2^. Nevertheless, hydrogen as a fuel is challenging to implement due to the difficulty to store, wide flammability and detonation range, ease to diffuse through numerous materials that could lead to the embrittlement in container metals^3^. Hydrogen is difficult to detect as it is colorless, tasteless and odorless^3^. Therefore, it is necessary to form sensors with high-sensitivity, low energy consumption and easy of fabrication^4^. Different gas sensors are mainly classified into optical, thermoelectrical, and conductometric ^5–7^. Nowadays, metal oxides are noticeable for sensing applications due to their crystalline size, physical, chemical properties^8–10^.

Tin oxide (SnO_2_) is a semiconductor material with n-type conductivity and a bandgap value of 3.7 eV, widely used as a sensing element^11^. However, some characteristics of SnO_2_, such us low selectivity, long response time and high operation temperature, restrict its successful application to hydrogen sensors. Many processes have been adopted to address these issues, such as surface passivation with noble metals (Platinum-Pt, Palladium-Pd)^12,13^. Notably, among the different deposition methods for SnO_2_, the sol–gel spin-coating process has many benefits for producing high-quality samples, including safe and straightforward fabrication with low-cost^14^.

In this work, we use density functional theory (DFT) to investigate the structural, electronic properties of the bulk and surface SnO_2_ and the changes occurring due to halogen, in particular, fluorine (F), chlorine (Cl), bromide (Br) and iodide (I), doping. Regarding the surface, we focus on the (110) facet and discuss the changes in the bandgap and the connection to the electrical conductivity from a theoretical viewpoint. Furthermore, we prepared five SnO_2_ thin-film samples, namely F:SnO_2_, Cl:SnO_2_, Br:SnO_2_ and I:SnO_2_ by using inorganic salts as solution precursors with the sol–gel/spin-coating method. The characterization of the crystalline structure and the electrical characteristics for the deposited thin films were analyzed using X-ray diffraction (XRD), atomic force microscope (AFM) and a four-point probe. We tested the sensing characteristics towards hydrogen gas of the doped SnO_2_ thin-films at room temperature. Here, we find that the room temperature response is highly improved when SnO_2_ is coated with just a small amount of Pt. The improvement of gas response caused by the addition of Pt- islands created on the surface can be attributed to one main reason: catalytic promotion obtained by the Pt particles, which also results to the formation of p–n junction between PtO_x_ and SnO_2_^15^. In this work, Pt particles are inserted in a small amount by using physical vapor deposition for 15 s. In the literature, many reports focus on the effect of fluorine doping in the electrical and optical properties of SnO_2_^16,17^. Specifically, fluorine dopant increases tin dioxide's conductivity and transparency, making it a better candidate for optoelectronic devices and gas sensors. In SnO_2_, it was previously shown that fluorine dopant occupies oxygen sites when inserted in low concentrations both in the bulk and the surface^18^. As fluorine has a higher electronegativity compared to oxygen^19^, the incorporation in the surface can attract more oxygen for the adsorption process and thus improve the response of the SnO_2_ sample towards hydrogen. Following this concept, halogen doping is a low-cost and easy to fabricate method to create more active sites for adsorbed oxygens or even hydrogen without significantly changing the properties of SnO_2_. Combining these characteristics with the band-structure manipulation, herein all the halogens are examined both in interstitial and substitutional position and then the sensing response towards H_2_ of the samples is discussed before and after doping. For the halogen dopants, NH_4_F, NH_4_Cl, NH_4_Br and KI were used as precursors, while SnO_2_ was fabricated using SnCl_2_ ˙ 2H_2_O.

## Theoretical simulations on halogen doped SnO_2_

### Structural properties

In the calculations a DFT a supercell consisting of 72 atoms is used. The experimental cell parameters are a = 4.737 Å, c = 3.142 Å^20^ and are in a good agreement with the calculated lattice parameters (a = 4.717 Å and c = 3.189 Å) In Figure S1 the simulated structure are examined. Specifically for the interstitial dopants, we explored many different configurations and we have chosen the minimum energy structure for our structural and electronic analysis. Using the geometry relaxation, we can also predict the lattice parameters of these structures shown in Table 1.

**Table 1:** The computed lattice parameters and cell volumes for every doping case.

	a(Å)	b(Å)	c(Å)	Vol ( Å^3^)
SnO_2_	4.717	4.717	3.189	70.956
F_i_:SnO_2_	4.730	4.715	3.193	71.210
F_o_:SnO_2_	4.729	4.729	3.202	71.613
Cl_i_:SnO_2_	4.784	4.748	3.200	72.650
Cl_o_SnO_2_	4.754	4.754	3.207	72.479
Br_i_:SnO_2_	4.784	4.783	3.195	73.106
Br_o_:SnO_2_	4.763	4.763	3.210	72.809
I_i_:SnO_2_	4.841	4.743	3.218	73.883
I_o_:SnO_2_	4.778	4.778	3.215	73.403

As it is shown in Table 1 and Fig. S2, the halogen dopants increase the volume of the supercell. To explain this result, we first focus on the substitutional case. The substitution of an oxygen atom with a halogen, which generally has a higher atomic radius value, increases the occupied space in the supercell and its volume. The same effect is observed in the interstitial position. As it is shown in Fig. S2, the interstitial doping repels the oxygen atoms thus increasing the volume of the supercell.

We use the formation energy to predict the energy cost to construct the halogen doped structures (*ΔΗ*_*f*_). The formation energy gives the energy requires to construct the structure by considering the chemical reservoirs of the structure. The formula for *ΔΗ*_*f*_ is:

1 $$\Delta H_{f}^{{\left( {D,q} \right)}} = \left( {E^{D,q} - E^{H} } \right) + \sum\limits_{i} {n_{i} } \cdot {\upmu }_{i} + q\left( {E_{f} + {\upvarepsilon }_{VBM}^{H} } \right)$$

where, *E *^*D,q*^ is the is the total energy of the defective supercell when the defect is in charge state *q*, *E*^*H*^ is the initial energy of the undoped host supercell, *μ*_*i*_ is the chemical potential of the atom removed or added. The free parameter in this equation is the *E*_*f*_ which represents the Fermi energy of the system with reference to the Valence band Maximum (VBM) which has energy ${\upvarepsilon }_{VBM}^{H}$ . All the these calculations are shown in Fig. S3. Table 2 shows the formation energy for neutral halogen dopants (q = 0). From these results, it is clear that at low doping concentration, the substitution of oxygen with halogens is more favorable compared to the interstitial incorporation. This has also been demonstrated experimentally^20^. There are not many reports regarding the formation energy of halogen dopants in SnO_2_. Williamson et al.^21^ predicted the formation energy of F:SnO_2_ at a value of 3 eV for the interstitial and 2.5 eV for the oxygen substitutional at 800 K and 1 atm environment. Behtash et al.^22^ examined the iodine doping of SnO_2_ and predicted that for the oxygen substitution the formation energy varies from 5 to 7.5 eV depending on the definition of the oxygen’s chemical potential. This value agrees well with the present prediction. Cheng et al.^23^ also worked on the halogen doping in SnO_2_ using the Perdew-Burke-Ernzerhof (PBE) functional for the structural properties and Heyd–Scuseria–Ernzerhof (HSE06) for the electronic calculations and his values agree well with the predicted formation energies.

**Table 2:** The formation energy of neutral halogen dopants in interstitial and oxygen position.

Defect case	Formation energy (eV)
F_i_:SnO_2_	1.20
F_o_:SnO_2_	0.46
Cl_i_:SnO_2_	5.10
Cl_o_SnO_2_	3.05
Br_i_:SnO_2_	5.97
Br_o_:SnO_2_	4.43
I_i_:SnO_2_	7.28
I_o_:SnO_2_	6.80

### Electronic properties of the bulk

**Figure 1:**
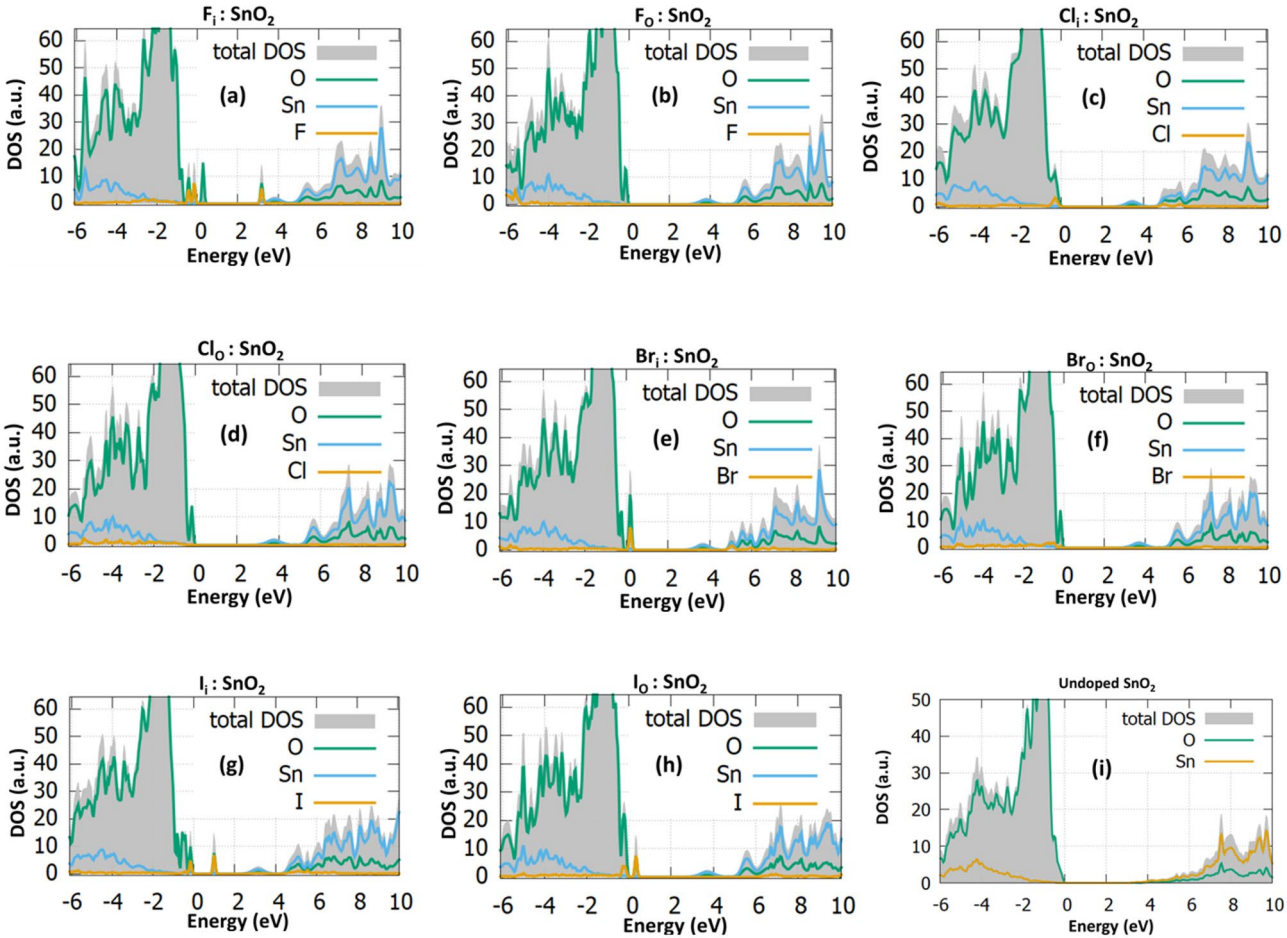
The projected DOS for the low concentration **(a)** F_i_:SnO_2_, **(b)** F_O_:SnO_2_, **(c)** Cl_i_:SnO_2_, **(d)** Cl_O_:SnO_2_, **(e)** Br_i_:SnO_2_ and **(f)** Br_O_:SnO_2_, **(g)** I_i_:SnO_2_, **(h)** I_O_:SnO_2_ and **(i)** Undoped SnO_2_.

To better understand the effect of halogen doping and the induced changes in the band-structure of SnO_2_ the density of states (DOS) is calculated before and after the halogen doping. The doping percentage is one halogen atom per 72 SnO_2_ atoms, which results in a 1.38% doping percentage. The electronic properties are calculated through the PBE0 exchange–correlation functional and the bandgap of SnO_2_ is computed to be 3.35 eV, in good agreement with the experimental band gap (3.7 eV^24^). This functional has also been used in other theoretical studies with similar value for the undoped case^21^. As it is presented in Fig. 1a, the fluorine interstitial in SnO_2_ gives rise to some additional energy states inside bandgap. These states originate by the hybridization of F-2p orbital with the nearest O-2p. The bandgap after the F_i_ incorporation is reduced to 3.00 eV. When F is inserted in O-substitutional position, additional states are created near the valence band maximum (Fig. 1b). Again, the bandgap is reduced to 3.00 eV. According to our calculations the bandgap value of the interstitial and substitutional is the same because the hybridization of the fluorine orbitals with oxygen orbitals in the interstitial case create energy levels inside the gap rather than in the band edges.

Regarding chlorine doping, the interstitial reduces the bandgap to a value of 2.54 eV (Fig. 1c) while gap states are created at the VBM. When Cl is at an O-substitutional position, Cl_O_, the bandgap reaches a value of 2.90 eV (Fig. 1d). Bromine interstitial intercalation results to a bandgap value equal to 2.91 eV (Fig. 1e). For the Br_o_ defect, the bandgap reaches a value of 2.84 eV (Fig. 1f). It is evident that regarding the electronic structure, Br doping has the same effect as Cl doping in terms of bandgap reduction.

Lastly, I interstitial and substitutional doping is examined. It is calculated that the bandgap is reduced to a value of 2.45 eV for the interstitial (Fig. 1g) and 2.75 eV for the substitutional case, respectively, while states near the valence band are again present (Fig. 1h). In this case, the interstitial doping has a smaller bandgap value than the substitutional case. This is attributed to the larger radius of iodine, compared to the other halogens which leads to a hybridization of the orbitals with its neighbouring atoms, when in an interstitial position, thus decreasing the bandgap more. This can be attributed to the states that are created to the valence band edge due to the hybridization of the 2p orbitals of the nearest oxygen atom with the iodine 5p orbitals. As iodine is larger than the other halogens, its orbitals are mixing with the nearest oxygen atoms, giving rise to the states near the valence band even in the substitutional position. These results indicate that halogen interstitial dopants serve as single acceptors when they are intercalated within the SnO_2_ host lattice. Such gap states near the oxide valence band maximum and those near the conduction band position may act as shallow and deep acceptors, respectively, hence contributing to performance enhancements of devices based on doped SnO_2_. In Fig. 1i the DOS of the undoped SnO_2_ is presented for reference. The changes in the bandgap can further boost the response of the gas sensor in the following way: a) The bandgap reduction helps the transition of charge carriers and thus it can be beneficial for the oxygen adsorption, b) The increase in Fermi energy affects the surface conductivity properties of the material, c) The mid-gap states can be beneficial for the sensing applications as they trap electrons which can be used from the adsorbed oxygen atoms and speedup the sensing process when the gas molecules are inserted. It is known that the gas sensing mechanism is highly connected with the trapped electrons, which mainly occur due to the gap states formed by the oxygen vacancies, as a result, it can be assumed that the combination of trap states due to halogen doping as well as the addition of more active sites due to the surface dopants can possibly improve the response towards a target gas. In Table 3 the bandgap values for the 1.38% as well as for the 2.08%^25^ are presented.

**Table 3:** The bandgap values of halogen dopants in interstitial and oxygen position at 1.38% and 2.08% concentrations.

Defect case	Bandgap of 1.38% concentration (eV)	Bandgap of 2.08% concentration (eV)^25^
F_i_:SnO_2_	3.00	3.10
F_o_:SnO_2_	3.00	2.90
Cl_i_:SnO_2_	2.54	2.85
Cl_o_SnO_2_	2.90	2.70
Br_i_:SnO_2_	2.91	2.90
Br_o_:SnO_2_	2.84	2.71
I_i_:SnO_2_	2.45	2.62
I_o_:SnO_2_	2.95	2.61

### Electronic properties of the surface

**Figure 2:**
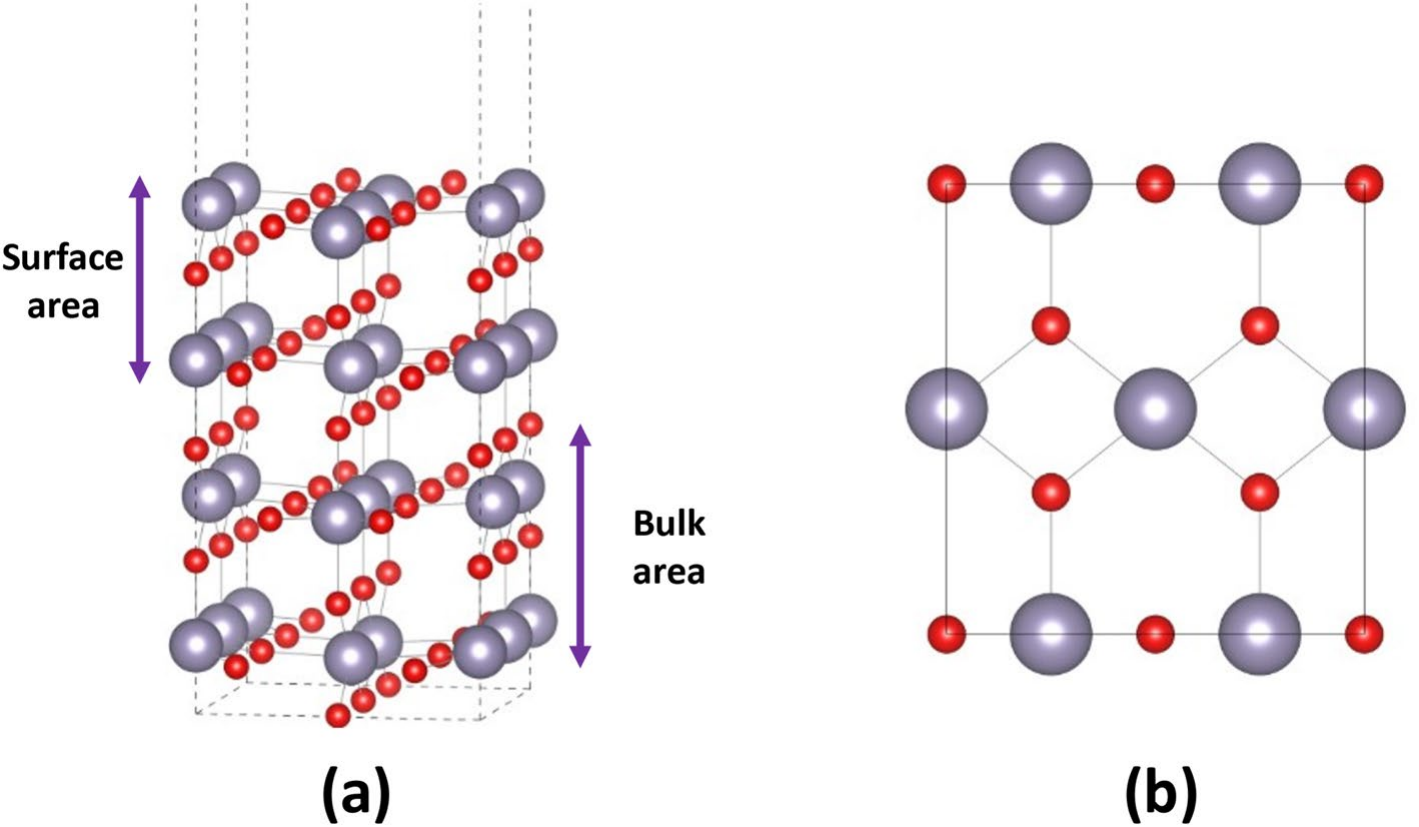
Representation of the SnO_2_ (110) Surface from **(a)** front and **(b)** top view.

To develop better SnO_2_ based sensing materials with high response and sensitivity, attention should be given on the surface doping. Theoretical simulations of the effect of surface doping on the electrical properties of SnO_2_ are less frequent in the literature, compared to the bulk system. In this section, attention is concentrated to the investigation of the electronic properties of the surface, before and after halogen doping. For the modelling of the surface a slab model with an (110) orientation is used, with a vacuum of 14 Å (see Fig. 2a,b). In the present model, the top 2 layers represent the surface and are fully relaxed while the bottom 2 layers are kept fixed as a representation of a bulk area. The perfect SnO_2_ (110) facet is the most stable low-index surface and it is a common choice for the simulation of the surface properties of SnO_2_^26^. The modelling of the surface is important as the gas adsorption and gas reactions take place mainly on the facets of the sample. Specifically, the changes of the surface bandgap and the surface band structure are highly connected with the changes in the electrical properties (for example, the conductance) due to halogen doping or gas exposure. As a result, mapping these changes not only in the bulk but also in the surface of the studied materials is worth mapping.

**Figure 3:**
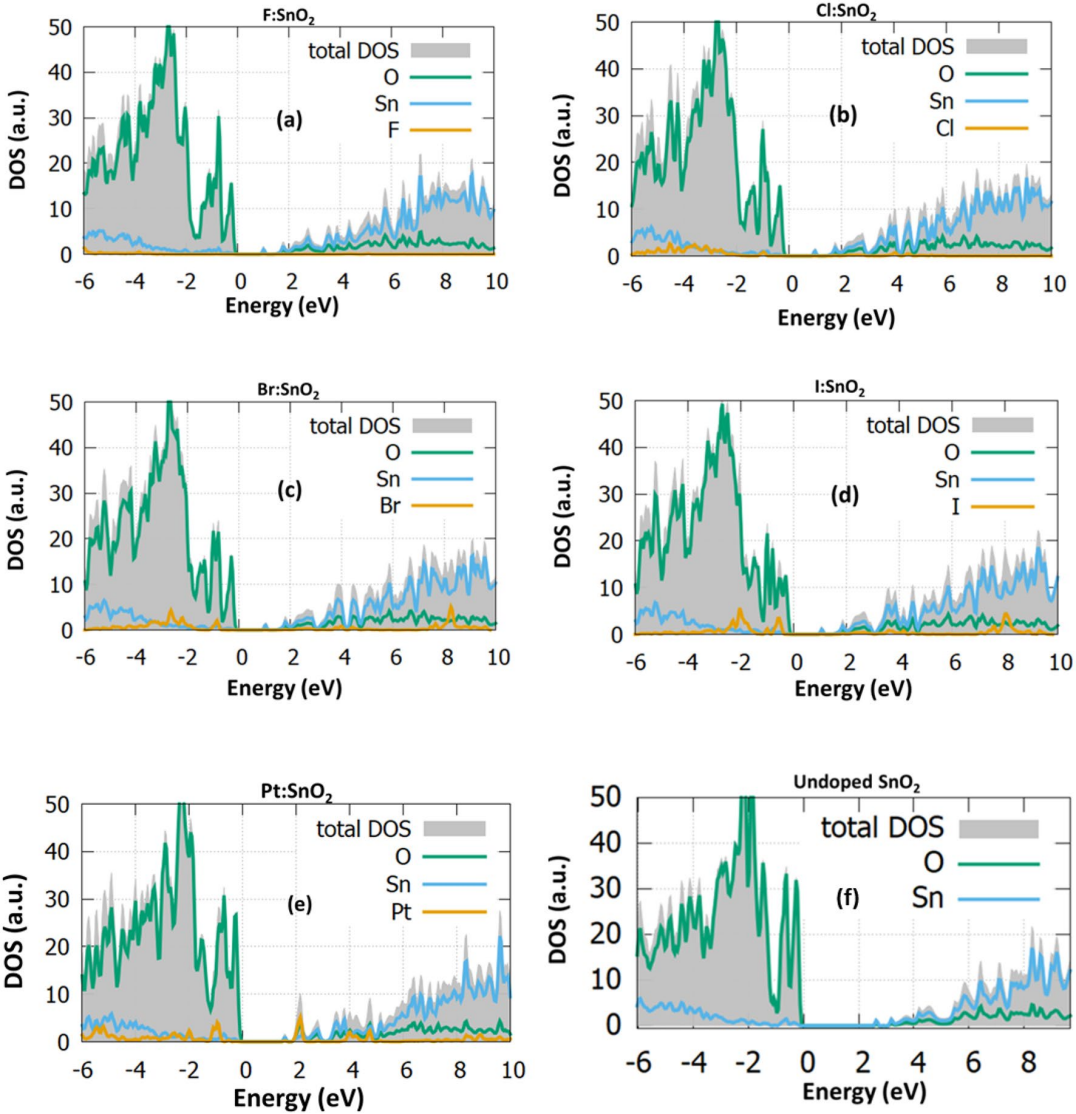
The electronic structure of **(a)** F:SnO_2_, **(b)** Cl:SnO_2_, **(c)** Br:SnO_2_, **(d)** I:SnO_2_ , **(e)** PtSnO_2_ and **(f)**Undoped SnO_2_.

The simulations reveal that the halogen dopants can be inserted in oxygen substitutional sites. In Fig. 3 the DOS for each supercell is shown and in Fig. 3f the DOS of the undoped is presented for reference. To accurate predict the electronic structure of SnO_2_ the hybrid functional PBE0 was employed. The bandgap value of the undoped surface is computed at 2.5 eV, in a good agreement with similar DFT works^27^. The smaller bandgap value compared to the bulk is attributed to the dangling bongs of the surface which create new states, responsible for this small reduction at the bandgap. Interestingly, in this high energy plane the bandgap value is decreased in all the halogen doping cases (Fig. 3a–d), reaching the value of 1.43 eV. All the above results indicate that halogen doping in SnO_2_ shows good characteristics for application in sensors. In Fig. 3e the Pt coating of SnO_2_ is investigated. Pt is generally used for the activation of the sensing process, but still the changes in the band structure have not been investigated. For this case, it is calculated that platinum reduces the surface bandgap and also intense energy states are created near the conduction band.

## Experimental investigation of halogen doped SnO_2_

### Structural characterization

To investigate the crystallinity of SnO_2_-based samples, XRD (Fig. S4) was employed in order to investigate the crystalline size (d) and the lattice parameters of the undoped SnO_2_. In the XRD spectra, diffraction peaks arise at 26.7°, 33.8°, 37^o^ and 52.2° which correspond to the (110), (101) and (200) facets. These peaks agree well with the reported peaks of the tetragonal rutile structure of SnO_2_^28^. Using Bragg’s law, the lattice parameters are determined, whereas with Scherrer’s equation the crystalline size of the samples. The lattice parameters were predicted according to the simulated peaks for each of the XRD spectra of the halogen doped structures. As it can be seen from the XRD, chlorine doped SnO_2_ exhibits the highest crystallinity while iodine doped SnO_2_ the lowest. Halogen doping produces shifts in all the facets, especially near 37.5 degrees. The lattice parameters where determined according to the shifted angles of each spectrum. The volume of Br:SnO_2_ is reduced in comparison to the undoped and to the relevent DFT result. This is because in the actual experimental conditions, intrinsic defects such as oxygen vacancies and tin interstitials are affected during the doping process. Consequently, the experimental results may vary from simulations. The results are presented in Table 4 and 5. In Fig. S5 the dependence of the volume with the dopant radius is presented and is in good agreement with the simulations. From the results in Table 4 we can conclude that in all the cases except I:SnO_2_ the average crystallite size is decreased. The reduction of the grain size to the nanoscale is one of the most efficient methods to enhance the response of the gas sensor. The thickness of the depletion layer that encapsulates the grains is constantly changing when the gas is inserted due to oxidation–reduction reactions^29^.

**Table 4:** The computed lattice parameters and cell volumes for every doping case.

	a(Å)	c(Å)	Vol ( Å^3^)
SnO_2_	4.720	3.170	70.623
F:SnO_2_	4.729	3.218	71.965
Cl:SnO_2_	4.734	3.222	72.207
Br:SnO_2_	4.723	3.207	71.537
I:SnO_2_	4.800	3.190	73.497

**Table 5:** The computed crystallite sizes for every doping case.

2θ	Planes	SnO_2_	F:SnO_2_	Cl:SnO_2_	Br:SnO_2_	I:SnO_2_
26.69	110	1.98	1.88	1.84	1.61	1.93
33.84	101	2.28	2.23	2.15	2.13	2.48
37.05	200	4.17	3.33	3.20	2.93	3.58
52.23	211	3.10	2.55	2.24	2.30	3.58

It is observed that the lattice constants for the undoped and halogen doped SnO_2_ have different values than those calculated with DFT. This is a common observation and it is associated with the intrinsic defects that highly affect the structural properties of the samples as well as the known limitations of the DFT. However, by applying DFT calculations, we can arrive at a good estimation and possibly reveal a trend of the effect of the addition of dopants in SnO_2_.

**Figure 4:**
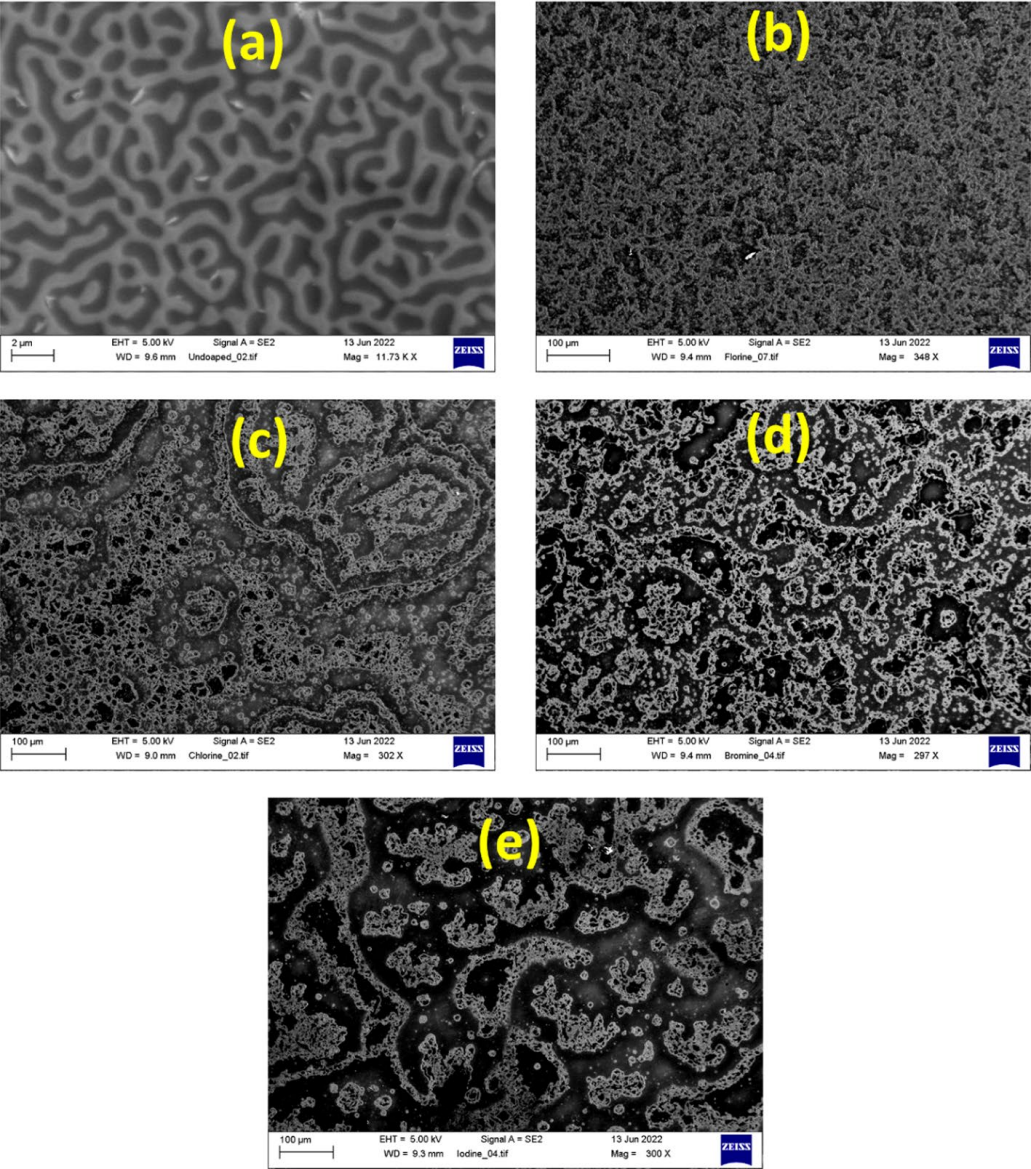
**(a)** The SEM images for the undoped sample, **(b)** F doped sample, **(c)** Cl doped sample, **(d)** Br doped sample and **(e)** I doped sample.

Figure S6 illustrates the AFM topographic images of the spin-coated pristine and halogen doped SnO_2_ while Fig. 4 shows the the SEM top-view images of the same samples. From the AFM images, a nanoripple morphology can be deduced for the pristine sample. The halogen doped samples maintain this morphology but with some noticeable differences compared to the reference sample. The root mean square (RMS) roughness is also calculated from AFM images. For the undoped SnO_2_ the roughness is predicted at 5.98 nm while it is 8.23 nm, 1.20 nm, 2.96 nm and 0.58 nm for F, Cl, Br and I respectively. Specifically with F doping, the roughness is increased compared to the undoped. The increase was not so sharp compared to the other halogens. We believe this is due to the radius of fluorine, which is close to oxygens. As a result, the incorporation of fluorine will not significantly change the structural properties, compared to the other halogens. This small reduction in roughness is possibly attributed to the incorporation of fluorine atoms in interstitial sites but still, further investigation is needed. The large decrease in the RMS roughness in the case of Cl, Br and I doped samples indicates that the dopant precursor salt is also incorporated inside the ripples thus filling the gaps between them. The morphology of the pristine and halogen doped samples are highly connected to the gas sensing properties of the materials as different roughness indicates changes in the active area which impacts on the current path and the depletion region. Furthermore, different surface is highly connected to the distribution of the Pt nanoparticles which affects the catalytic activity on the sample^30^. SEM images (Fig. 4), also reveal a wrinkle network structure that consists of dense grains from agglomerated nanoparticles with narrow particle size distribution. These nanoparticles were self-assembled to produce nanoporous wrinkle network structures with pores. After halogen doping, these networks are seen to increase for the case of F, Cl and Br but for I they become narrow. As a result, the particles that constitute these films are increased per unit area, giving more sites for the sensing reaction to occur. So, from a morphological perspective, halogen doping is appropriate for many nanotechnological applications such as sensors and photocatalysis.

### Electronic structure and bandgap

For the investigation of the bandgap changes of our samples, the optical properties are probed through the Ultraviolet–Visible (UV–Vis) spectroscopy. As it is presented in Fig. S7a, the UV–Vis absorption spectra reveal that all samples have an absorption range in the ultraviolet light region. Compared to the undoped SnO_2_ sample, the halogen-doped structures have an increased UV light absorption with the iodine doping showing the highest absorption. Halogen doping actively enhances the visible light absorption of SnO_2_, which is consistent with other reports^31–33^. Fluorine doping is seen to increase the bandgap of SnO2 slightly. This is opposite to what was predicted from our DFT calculations and can be attributed to the synergetic effect of fluorine interstitials and substitutionals as well as the combined effect of the intrinsic defects of SnO_2_. In the case of I:SnO_2_, however, it is observed that even though enhanced absorption within the visible is achieved relative to the pristine sample, highest concentration of iodine reduces the visible absorption of the I doped sample. This is probably an indication that during high halogen doping, some of the intercalated I atoms have been inserted in interstitial sites, producing mid-gap states that reduce the transition of electrons from valence band to conduction band thus reducing the absorption of the material. The band gap value of each sample was calculated from the corresponding Tauc plots, which are presented in Fig. S7b. It is clear that the bang gap for the halogen doped samples is near the gap of pure SnO_2_. In the case of I:SnO_2_, the band gap is highly reduced, which is in agreement with the previous DFT calculations. From the above, it is demonstrated that halogen treatment can improve the absorption capacity of SnO_2_. Table 6 summarizes all the band gap values. This decrease of the bandgap due to halogen doping can be beneficial for other applications apart from the gas sensors. Specifically, the improvement in the absorption of Br:SnO_2_ and I:SnO_2_ can be used in applications such as hydrogen production while the increase in transparency due to fluorine doping can be used in applications such as organic and perovskite photovoltaics, where the SnO_2_ is used as electron transport layer (ETL) deposited on the transparent bottom electrode. The bandgap reduction can also be beneficial for the response in gas sensors. Specifically, reduced bandgap is translated in easier transition of an electron from the valence band to the conduction band. So it is possible that the reduce bandgap can help the capture of electrons from the adsorbed oxygen species and also can help the transition back to the conduction band after the gas sensing reaction between the oxygens and the target gas.

**Table 6:** Band gap values for every halogen doping case.

Halogen dopants	Band gap (eV)
F	3.52
Cl	3.43
Br	3.46
I	3.36
Undoped	3.50

Changes in composition upon doping are also investigated by using X-ray photoelectron spectroscopy (XPS) measurements. Moreover, XPS is an sensitive and accurate method to experimentally reveal if the halogen doping is achieved and at what quantities the dopants are inserted. Figure S8a presents the XPS spectra of the F 1 s region for the F doped samples. For the F:SnO_2_ sample, two peaks can be seen at 688.3 and 684.4 ± 0.1 eV attributed to F-Sn–O bonds and F^-^ adsorbed on the surface, respectively^35^. This was also the case for the Cl 2p region for the Cl doped samples as shown in Fig. S8b. Traces of chlorine were found at all the samples because it is an element, contained to the precursor. Considering the Br doped sample, the Br 3d region is presented in Fig. S8c, where a single peak can be seen at 69.4 ± 0.1 eV. Finally, the results for iodine are presented in Fig. S8d. The XPS results show that the dopant percentage varies from 1 to 2% on the SnO_2_ surface. As a result, the experimental results are in a good agreement with the DFT simulations which use 1.38% dopant.

**Figure 5:**
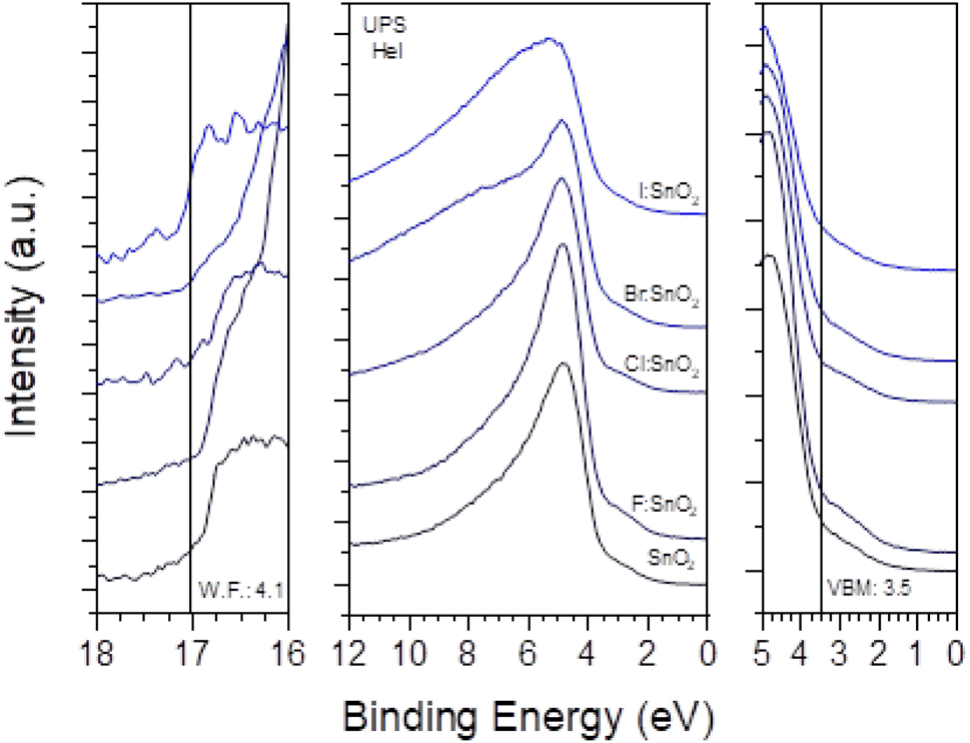
The UP spectra for the halogen doped samples.

In Fig. 5, the UPS (middle), the high Binding Energy (BE) cut-off (left) and the VBM (right) regions are presented for the doped and undoped SnO_2_ samples. Considering the work function (W_F_), as taken from the high BE cut-off, for the pristine SnO_2_ sample, is 4.1 ± 0.1 eV^36^ and a slight decrease, up to 3.9 ± 0.1 eV, as the atomic number of the halogen increases^37^. This decrease in the W_F_ indicates the n-type doping character of the halogen incorporation within the SnO_2_ which can contribute to enhanced electron conductivity. The VBM for all the samples is measured from the intersection of the Valence Band cut-off with the background and is 3.5 ± 0.1 eV^38^. The shoulder present around 2.8 eV is due to gap states induced by the lone pair Sn 5 s states. Compared to the DOS calculations in Fig. 1 it is observed that the CB has a similar form in all the halogen doped cases.

## Electrical characterization and gas response

To investigate the effect of temperature to the electrical properties of halogen doped SnO_2_ devices, the initial resistance values of the prepared SnO_2_ samples at different operation temperatures were determined. As it is shown in every examined case in Fig. S9, the conductance of SnO_2_ increases with temperature. The reason for this observation, is the ability of SnO_2_ to adsorb oxygen molecules with the increasing temperature. Considering that the chemisorbed atomic oxygens release electrons when the hydrogen gas is introduced, this can impact the sensing characteristics of SnO_2_. The present results agree with similar studies with Chemical Vapour Deposition (CVD) SnO_2_ and other similar sol–gel thin films^8^^,^^39^. From Fig. S9b–e, for the halogen doped SnO_2_ samples, when the temperature returns to the initial point, the resistance changes compared to the initial value. This can be attributed to the kinetics of the dopants due to the variation of the temperature or the structural changes occurring. This can be considered as a disadvantage of the halogen doping compared to the undoped, as it is seen that every thermal cycle produces different results.

**Figure 6:**
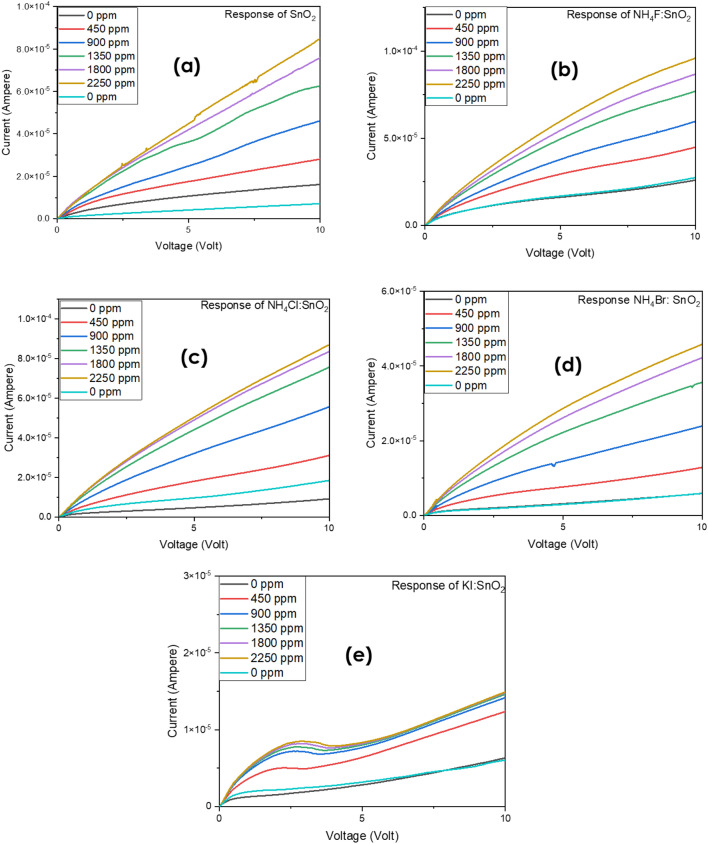
The response of **(a)** pristine SnO_2_, **(b)** F-doped, **(c)** Cl-doped, **(d)** Br-doped and (e) I-doped SnO_2_ based samples in room temperature.

**Figure 7:**
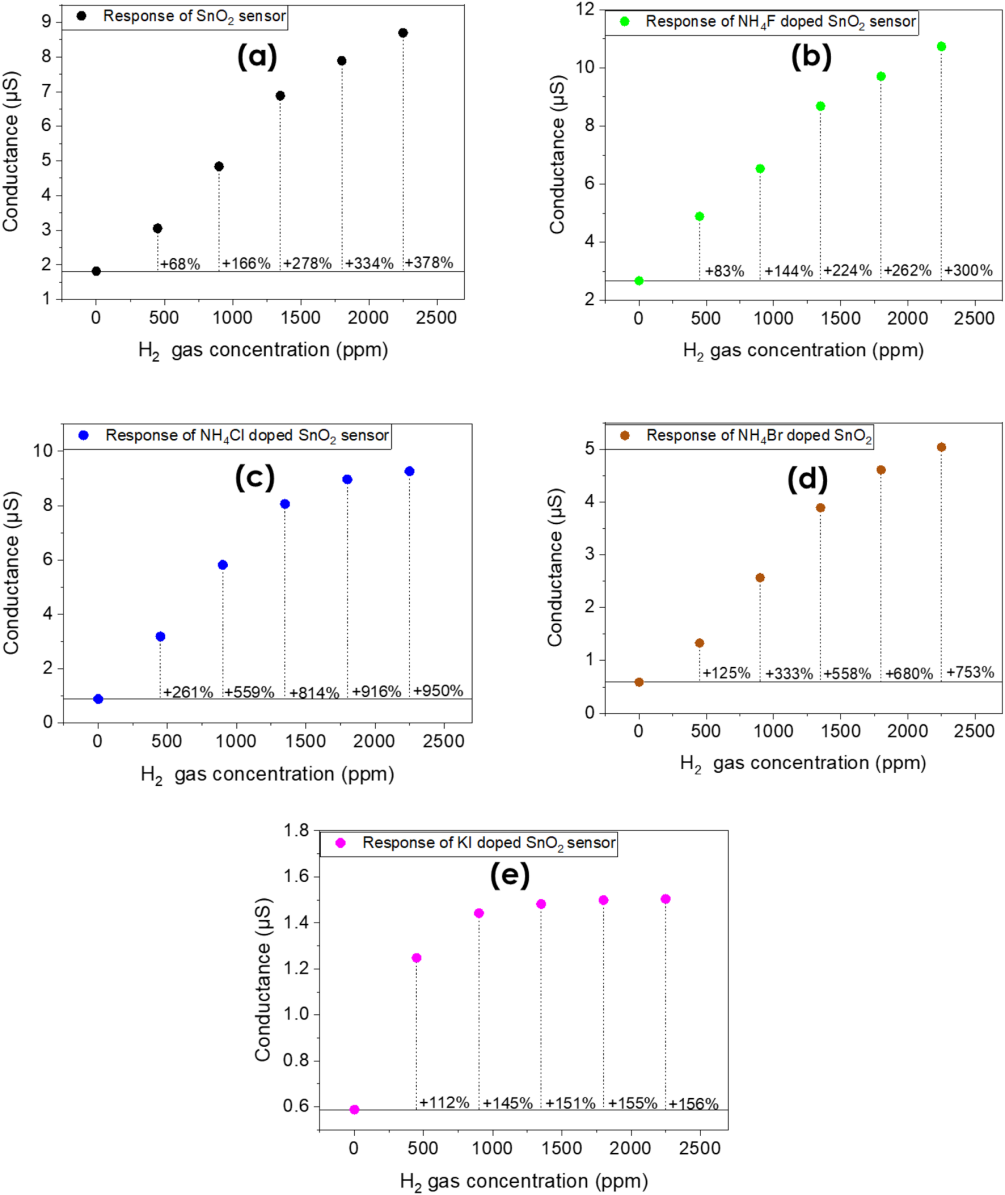
The response of **(a)** pristine SnO_2_, **(b)** F-doped, **(c)** Cl-doped, **(d)** Br-doped and (e) I-doped SnO_2_ based samples in room temperature for a fixed voltage value equal to 5 V.

The response of the fabricated sensors based on the halogen doped and undoped SnO_2_ upon different hydrogen concentration was also investigated. Figures 6 and 7 present the current–voltage (I-V) characteristic curves of the devices when different quantities of hydrogen inserted in the measurement’s system. As it is shown, every SnO_2_ sample increases its conductivity when the inserted gas is increased. This result agrees well with previous experimental studies^40^. From the present experimental results it is seen that halogen doping highly improves the response of tin dioxide especially in the case of Cl:SnO_2_ achieving the highest response in room temperature. This can be attributed to the reduced grain size of the surface as well as the reduced bandgap value. Furthermore, it is possible that the gap states that were created due to halogen doping might work as traps for the charge carriers and thus improving the response of the sensor. These results are summarized in Table 7. The sensing mechanism of the halogen doped SnO_2_ thin films is as follows: when we expose the surface of doped SnO_2_ to air ambient, the oxygen species will be absorbed, while when the surface is in a H_2_ gas environment there is a reduction of the exposure gas by the adsorption of oxygen molecules on the surface which this will increase the conductivity upon exposure. Depending on the operating temperature, the adsorbed oxygen species will capture electrons from the nanocrystalline halogen doped SnO_2_ thin films surface and become negatively charged which will increase the depletion region and therefore increase the resistivity^39^. From our research we noticed that at higher doping concentrations (as for example for 700 μl dopant in 700 μl SnO_2_) the current voltage measurements remain un-changed with the hydrogen insertion. We believe that this happens because when the halogen dopants are in higher concentrations, they tend to sit in oxygen vacancies sites. The reduction of oxygen vacancies is translated to reduction in adsorbed oxygens and thus the response of the sample is significantly reduced.

**Table 7:** Response/ Sensitivity with respect to hydrogen concentration of the undoped and halogen doped SnO_2_ thin films.

Gas Concentration (ppm)	SnO_2_ (%)	F:SnO_2_ (%)	Cl:SnO_2_ (%)	Br:SnO_2_ (%)	I:SnO_2_ (%)
450	68	83	261	125	112
900	166	144	559	333	145
1350	278	224	814	558	151
1800	334	262	916	680	155
2250	378	300	950	753	156

From the presented results it can be seen that at low concentrations, halogen doping is beneficial for improving the response towards hydrogen gas. At higher concentrations, fluorine doping is seen to reduce its performance compared to the undoped sample. From our understanding this could be happening because at high gas concentrations hydrogen molecules possibly change the amount of surface F as they interact with it. By changing the number of surface F atoms it is possible that the number of active sites is reduced for the sensing reaction to take place, and thus the response is reduced. Our proposed mechanism for the improvement of the response towards hydrogen is as follows: It is possible that halogen doping increases the surface oxygen vacancies which work as active sites for adsorbed oxygen from air and this enhances the response of the sensor. Moreover, it is possible that the halogen molecules of the surface can be themselves active sites and interact with the inserted hydrogen molecules. Apart from the oxygen vacancies, other intrinsic defects possibly affect the performance of the gas sensor but it is not in the scope of this paper to investigate this phenomenon.

The response of halogen doped SnO_2_ devices is comparable to other reported sensors which operate at higher temperatures. Pippara *et al.*^41^ fabricated an SnO_2_ gas sensor doped with polyaniline and palladium that operated at room temperature and had a maximum of response equal to 540%. This result was achieved at 400 ppm. Compared to the Cl:SnO_2_ device, this result is better for low concentrations. However, at high gas concentrations the chlorine doping is more beneficial. Furthermore, doping with palladium is of higher cost compared to doping with chlorine or the other halogens. A similar undoped SnO_2_ sensor was fabricated from Tounier and Pijolat^42^ were they predicted that at 500 ppm the response of SnO_2_ reaches a value of 170% at operating temperature of 500 °C. This result was better compared to all the halogen doped cases examined in this paper except from the chlorine doping which still achieves higher sensitivity. Kadhim and Hassan^43^ fabricated an undoped SnO_2_ gas sensor which performed with a response of 600% at room temperature for 1000 ppm of H_2_. Although this performance is better compared to the undoped that is fabricated in this work, still the Cl:SnO_2_ shows better results. From all the above, it is seen that halogen doping and especially chlorine is a promising and low-cost method to fabricate portable devices that work beneficially at room temperature.

## Conclusion

In this paper the interaction of halogen doped SnO_2_ with hydrogen both from theoretical and experimental point of view is extensively investigated. DFT is used to investigate the changes in the structural, thermodynamical, and electronic properties due to halogen doping. It was found that halogen doping creates gap states that are beneficial for the adsorption of oxygen and hydrogen which are responsible for the change of the resistance of the sample. The incorporation of halogens is most probably occurred in substitutional positions while the iodine requires a higher amount of energy to be inserted in the structure. Regarding the experimental part, low-cost and easy-to-fabricate devices are created. Halogen doping is an easy method to enhance the response of SnO_2_ device in room temperature conditions. The Cl:SnO_2_ sensors show better response at every gas concentration at room temperature. The results towards H_2_ are comparable to the results of SnO_2_ in much higher operating temperatures. This type of enhancement in room temperature conditions is reported for the first time and can be practically used for the detection of dangerous gases. Using advanced characterization techniques we found that halogens reduce both the bandgap and the work function of SnO_2_. Through these experiments it was found that many or the electronic and optical characteristics are improved due to halogen doping; we argue that this improvement could be beneficial for other applications such as hydrogen production or photovoltaics. Although this work preliminary studies the properties of halogen doping as well as the response towards hydrogen, further experiments are required to investigate the other parameters of the gas sensors as well as the exposure to other gases apart hydrogen.

## Supplementary Information

Supplementary Information.

## Data Availability

The datasets used and/or analysed during the current study available from the corresponding author on reasonable request.
