# Supplementary material for: Optimization of the hydrogen response characteristics of halogen-doped SnO2
Source: Sci Rep. 2023 Feb 13;13:2524. doi: 10.1038/s41598-023-29312-6 (PMC9925754; doi:10.1038/s41598-023-29312-6)
Supplement: Supplementary file 1 — Supplementary Information. [file 41598_2023_29312_MOESM1_ESM.docx]

**Optimization of the hydrogen response characteristics of halogen-doped SnO_2_**

Petros-Panagis Filippatos ^1,2,*^, Rohit Kumar Sharma ^2^, Anastasia Soultati ^1^, Nikolaos Kelaidis ^1^, Christos Petaroudis ^1,3^, Anastasia-Antonia Alivisatou ^4^, Stella Kennou ^5^, Stavros-Richard G. Christopoulos ^2^, Dimitris Davazoglou ^1^, Maria Vasilopoulou ^1,^, Alexander Chroneos ^6,7,*^

*^1^Institute of Nanoscience and Nanotechnology (INN), National Center for Scientific Research Demokritos, 15341 Agia Paraskevi, Athens, Greece*

*^2^Faculty of Engineering, Environment and Computing, Coventry University, Priory Street, Coventry CV1 5FB, United Kingdom*

*^3^Department of Electrical and Electronics Engineering, Faculty of Engineering, University of West Attica, Campus 2, No. 250, Thivon str. 12244, Athens, Greece*

*^4^ School of Mining and Metallurgical Engineering, National Technical University of Athens, 9 Iroon Polytechniou Str., Zografou Campus, Athens 15780, Greece*

*^5^Department of Chemical Engineering, University of Patras, 26504 Patras, Greece*

*^6^ Department of Electrical and Computer Engineering, University of Thessaly, 38221, Volos, Greece*

*^7^Department of Materials, Imperial College, London SW7 2AZ, United Kingdom*

1. **Methodology**

For all the computational simulations the Cambridge Serial Total Energy Package was used ^1^. For the relaxation of the examined structures the PBE functional is used ^2^ while for the electrical properties the hybrid PBE0 functional is employed with which the underestimation of the bandgap is encountered due to the localized electrons ^3^. A supercell consisting of 72 atoms was used. For the sampling of the Brillouin zone for the DOS calculations a k-point mesh of 4x4x4 points is chosen. The doped SnO_2_ samples were deposited by using spin-coating method for the SnO_2_ solutions. For the preparation of the undoped samples 0.045g SnCl_2_ ^.^ 2H_2_O in 2ml absolute ethanol is dissolved. The solution was continuously stirred for 24h at room temperature at speed 500 rpm. The sensor devices were deposited on Si substrate as follows: The solution was coated on Si at the angular speed of 3000 rpm for 30s and then the substrate was dried at 110^o^C for 10min. This procedure was repeated for 10 times. After the ten layer deposition the sample was left to crystallize at 500^o^C for 2h. Lastly, the e-beam evaporation technique to deposit a thin layer of Pt on SnO_2_ as a catalyst is used. For the halogen dopants, 10mg of NH_4_F, NH_4_Cl, NH_4_Br and KI precursor was mixed with 1mL water. The doped structures were formed by mixing 70μl dopant with 700 μl SnO_2_. X-ray diffraction (XRD) measurements were carried out with a Siemens D500 diffractometer with Cu-Ka radiation. A Perkin Elmer Lambda 40 UV–vis spectrometer was used to record the absorption spectra of the samples. All the samples were introduced to the ultra-high vacuum chamber for XPS and UPS measurements as received. XPS measurements were performed using unmonochromatized Al K*α* X-rays (1486.6 eV) and a hemispherical energy analyzer (Leybold EA-11) with a constant pass energy of 100 eV. The full width at half maximum for a reference Au 4f_7/2_ peak is 1.5 eV. The analyzed area was an approximately 2 × 5 mm^2^ rectangle positioned near the geometric center of each sample. XPS analysis was carried out at 0° take-off angle (normal to the sample surface). The C 1s peak at 284.8 eV binding energy was used for surface electrostatic charging correction in all spectra. For the UPS measurements, He I line was used and a bias was applied between the sample and the analyzer in order to separate the secondary electrons.


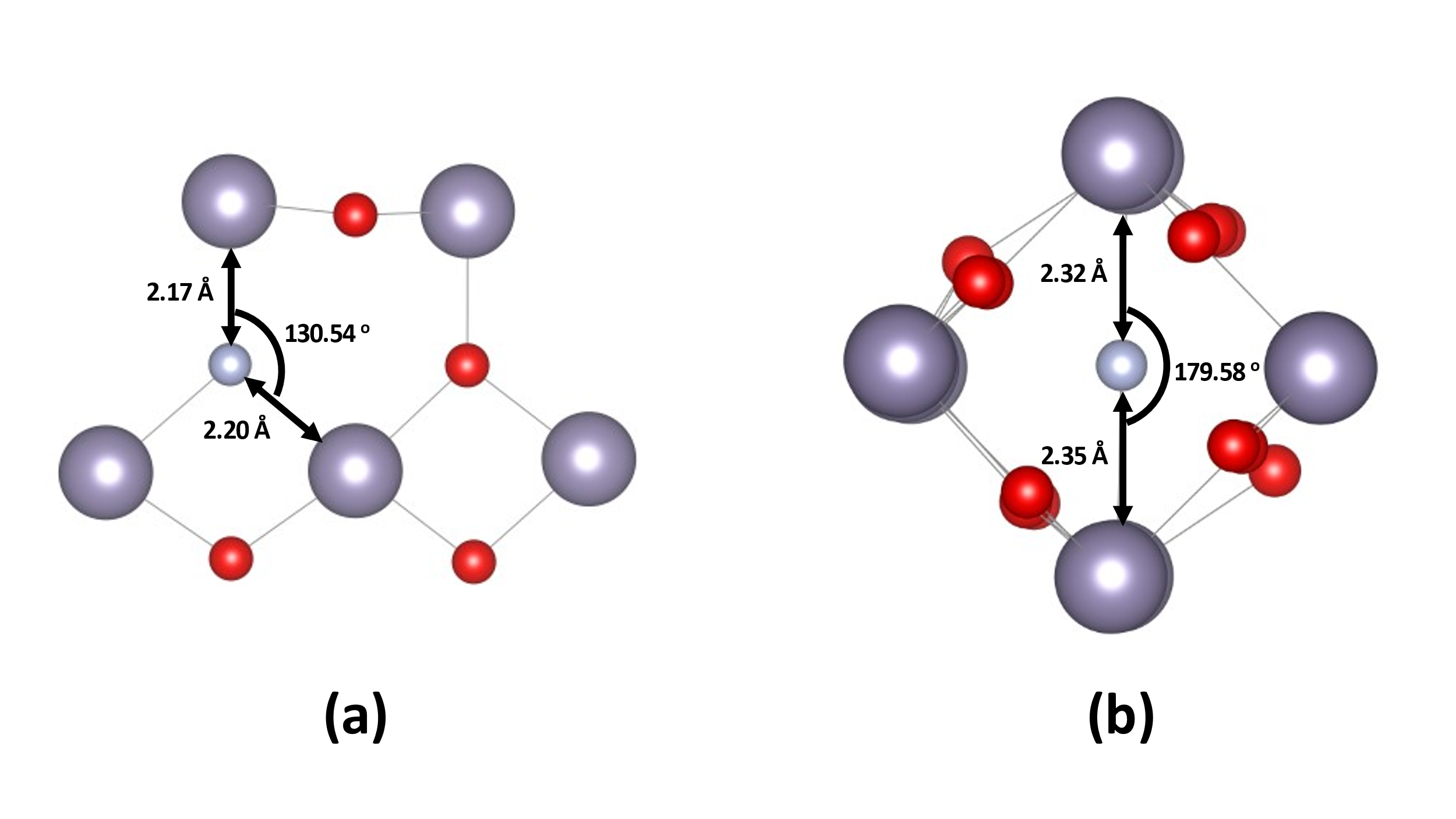


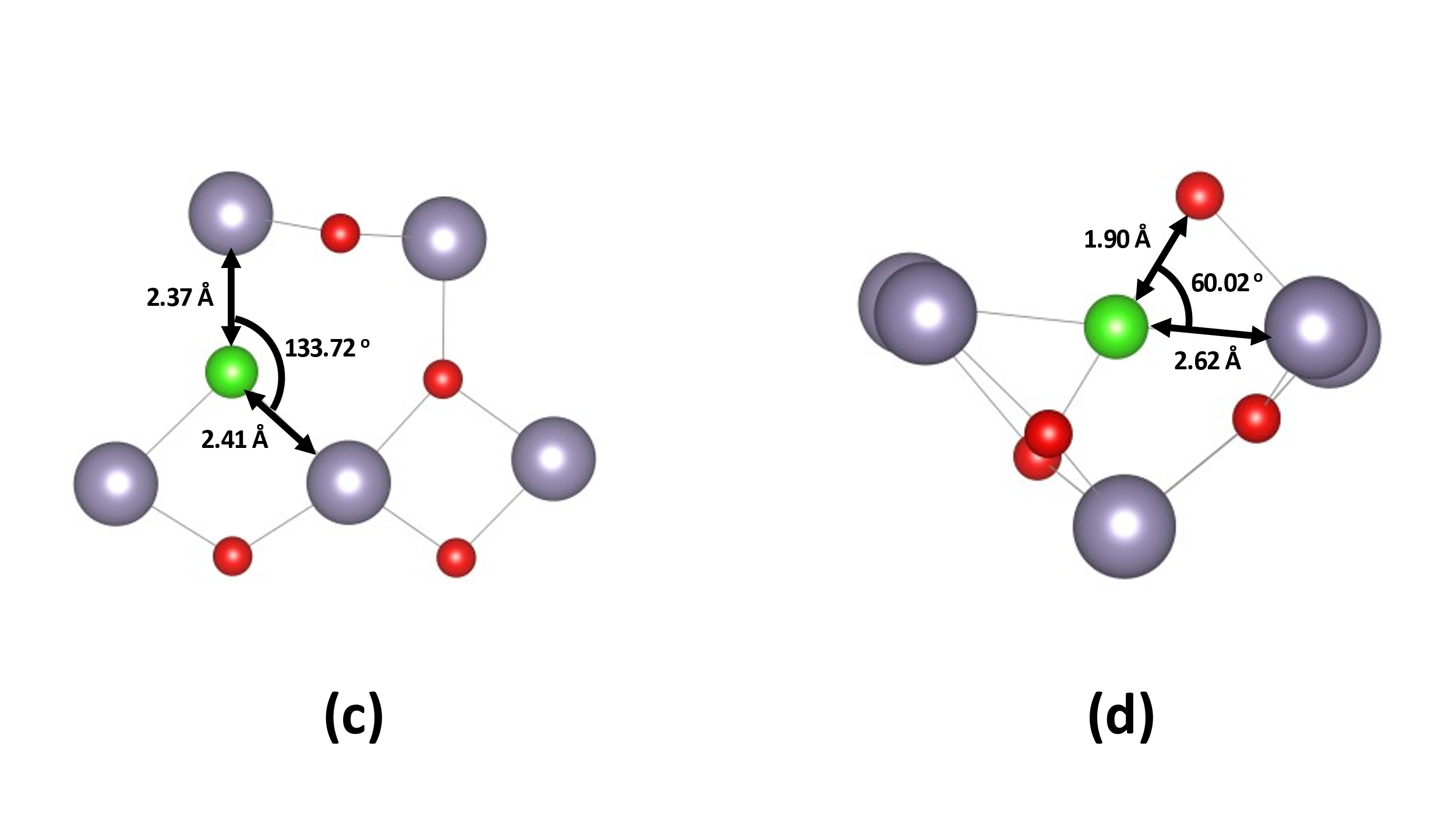


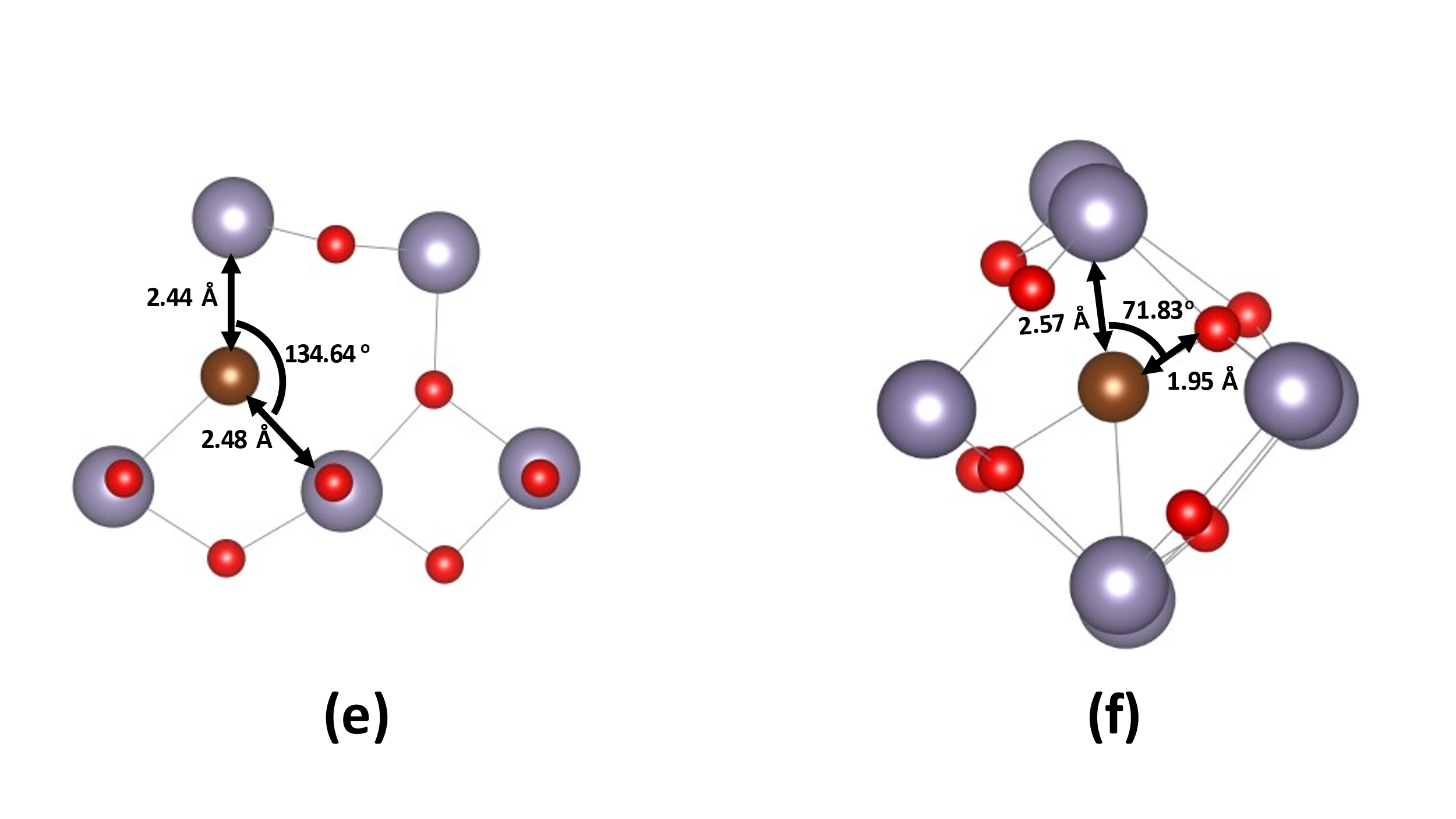


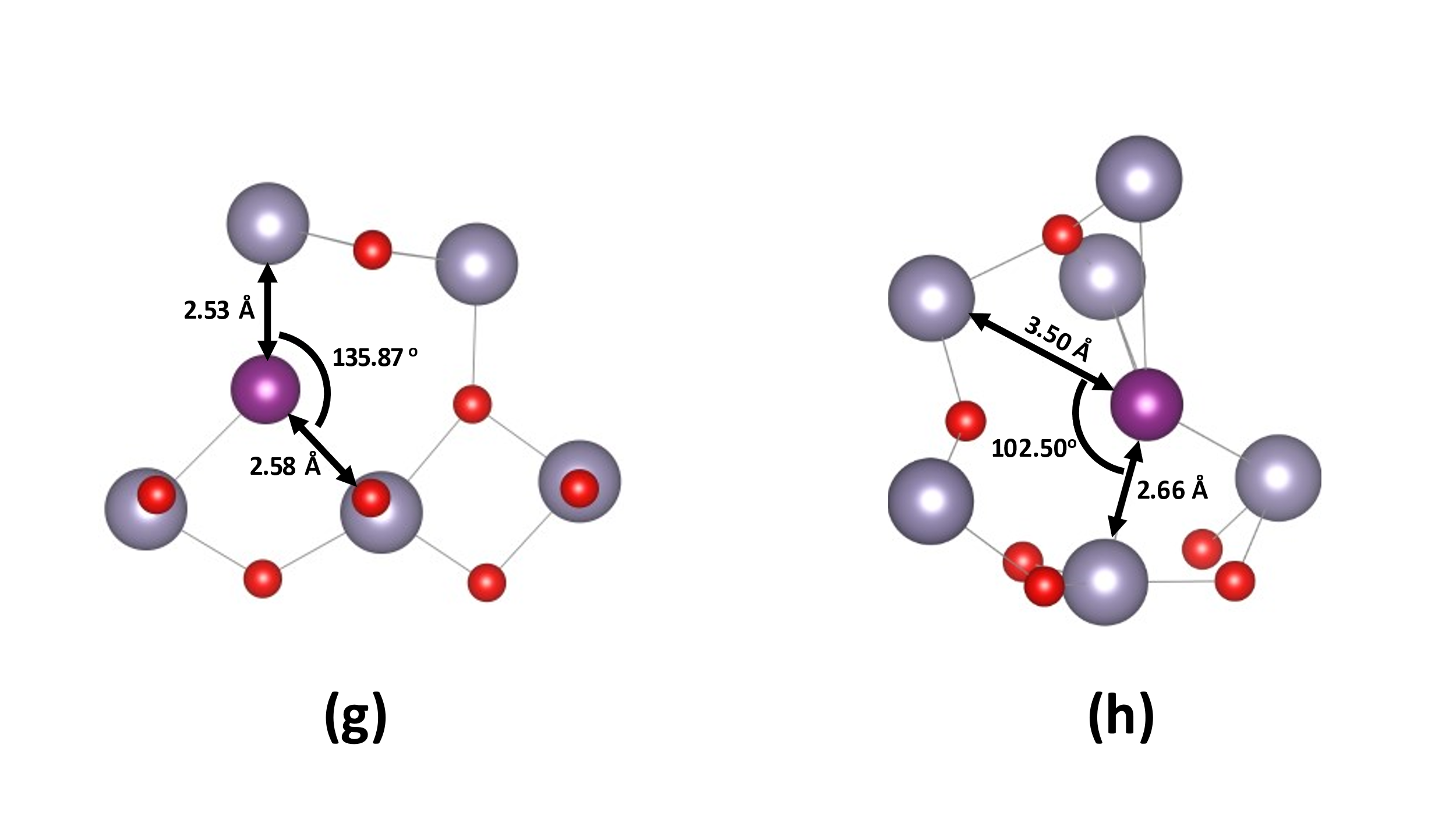


***Figure S1.*** *The optimized structures of (a) F_o_ :SnO_2_ ,(b) F_i_ :SnO_2_, (c) Cl_o_ :SnO_2_, (d) Cl_i_ :SnO_2_ , (e) Br_o_ :SnO_2_ , (f) Br_i_ :SnO_2_ , (g) I_o_ :SnO_2_, (h) I_i_ :SnO_2_.*

**
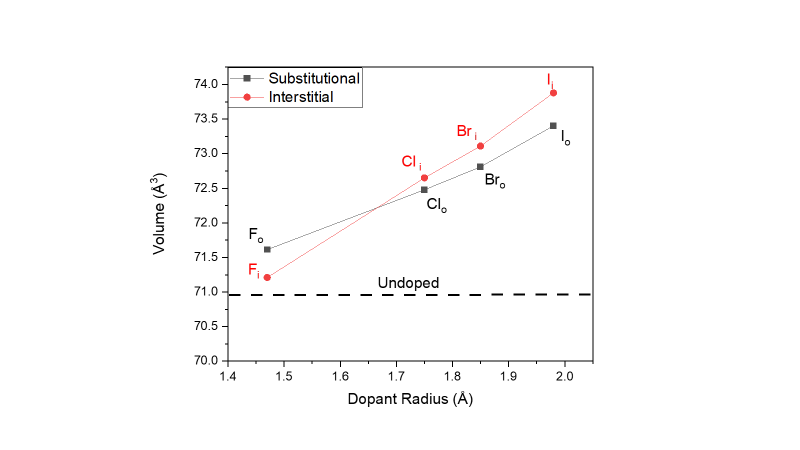
**

***Figure S2.*** *The volume for every halogen dopant for* *substitutional and interstitial incorporation.*


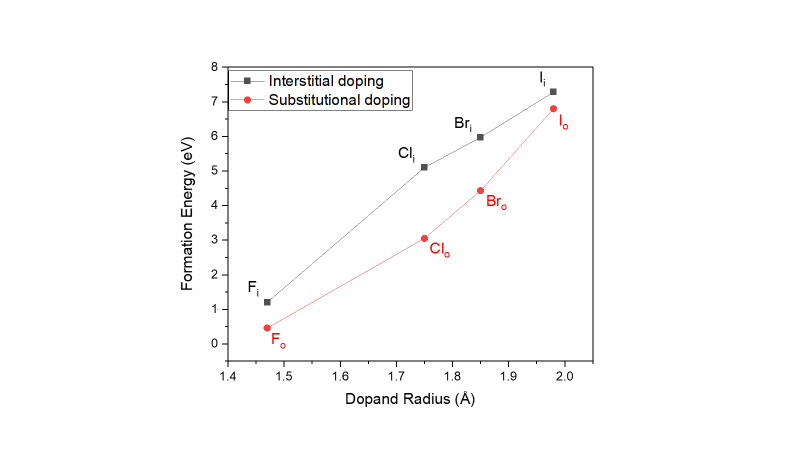


***Figure S3.*** *The halogen formation energy for substitutional and interstitial incorporation.*


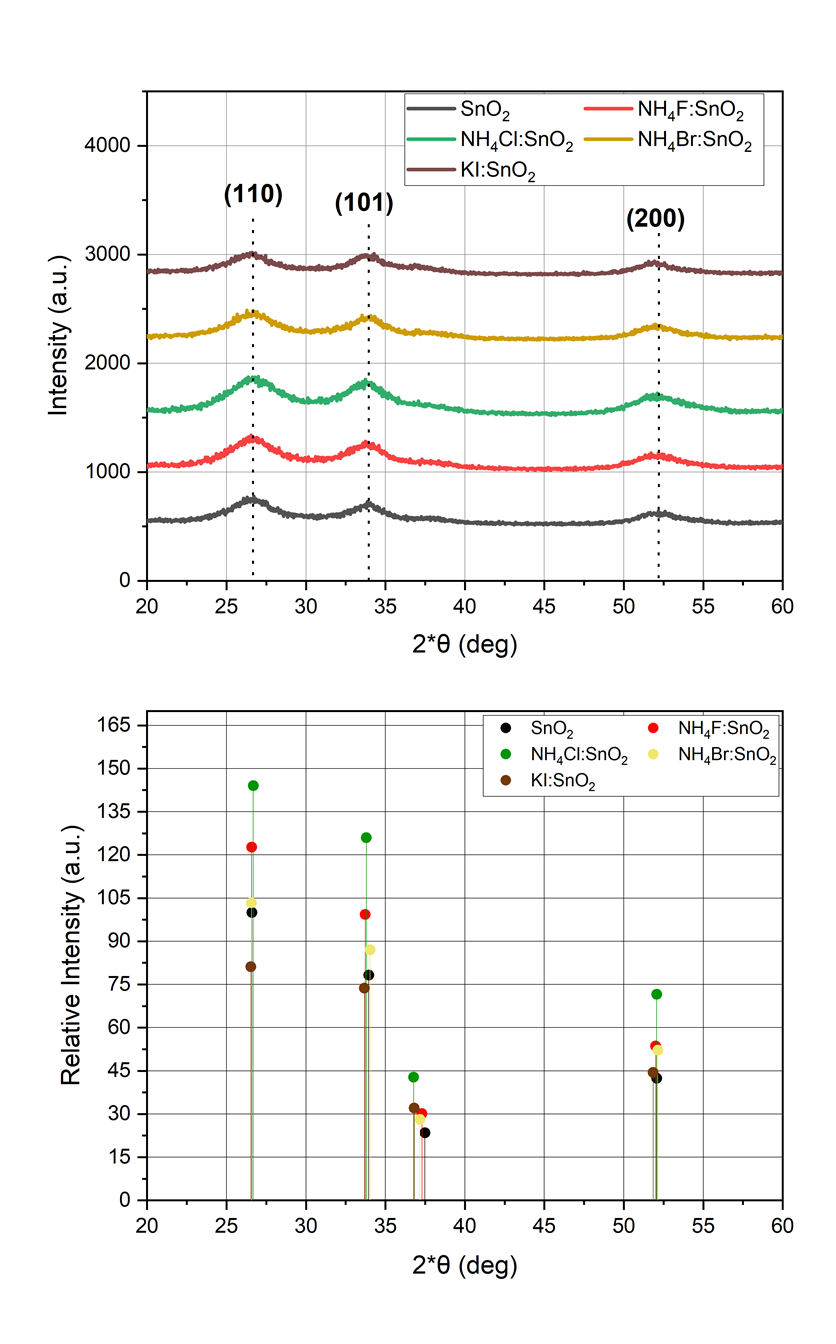


***Figure S4.*** *The XRD plots for the undoped and doped structures.*

***
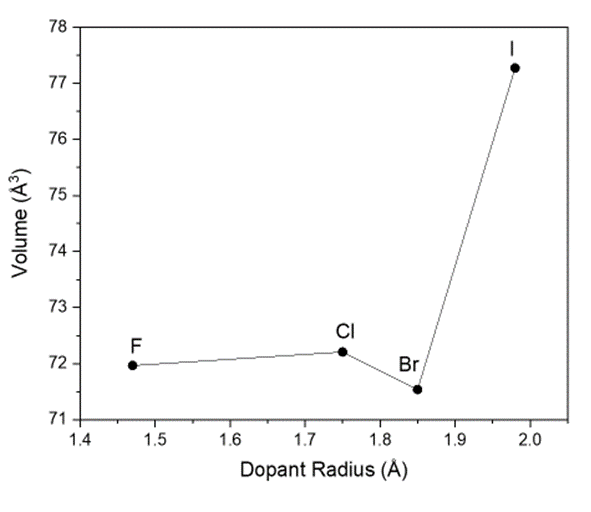
***

***Figure S5.*** *The dependence of dopant radius with the volume of the unit cell.*

| ***SnO_2_*** | 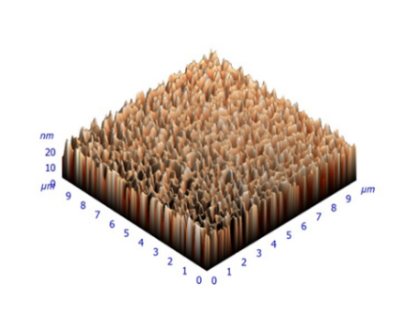 | 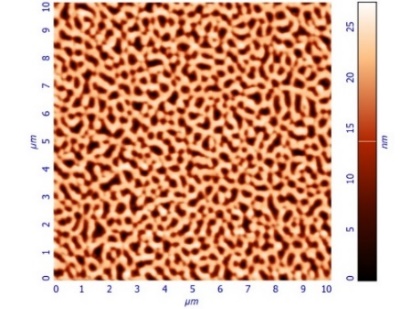 | 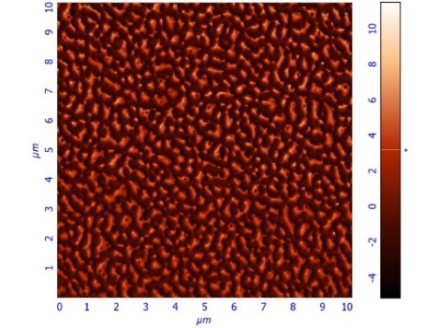 |
| --- | --- | --- | --- |
| ***F:SnO_2_*** | 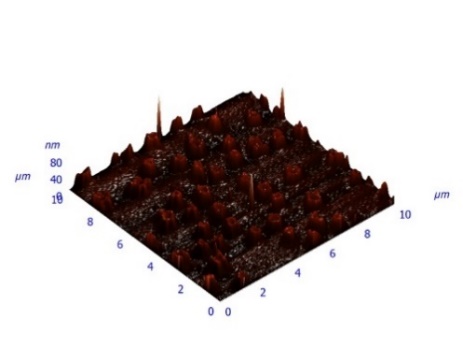 | 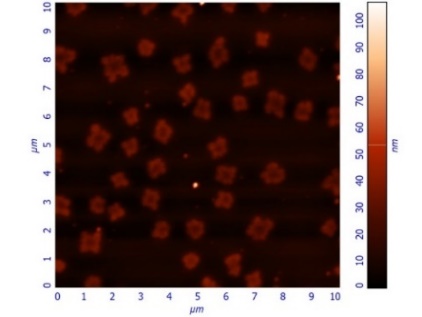 | 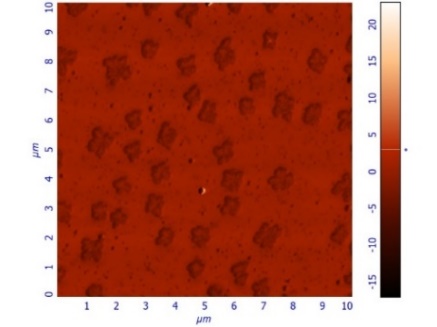 |
| ***Cl:SnO_2_*** | 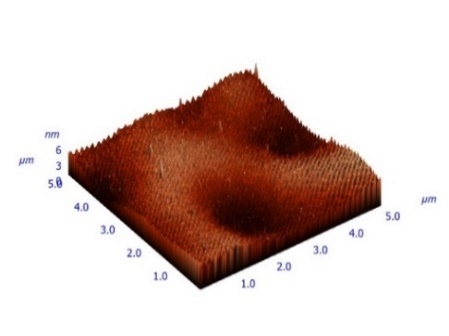 | 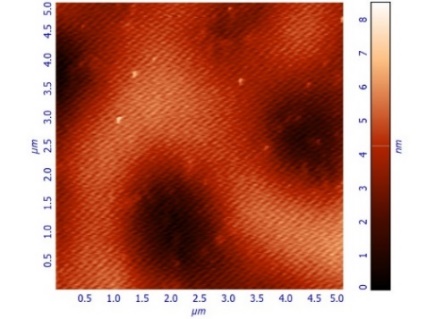 | 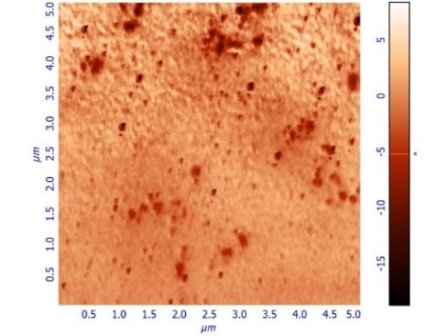 |
| ***Br:SnO_2_*** | 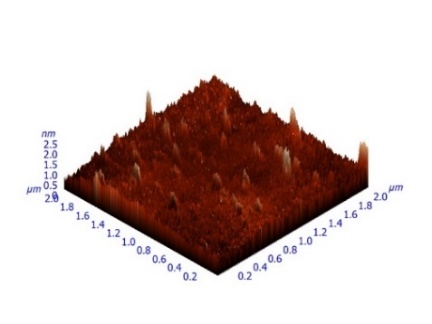 | 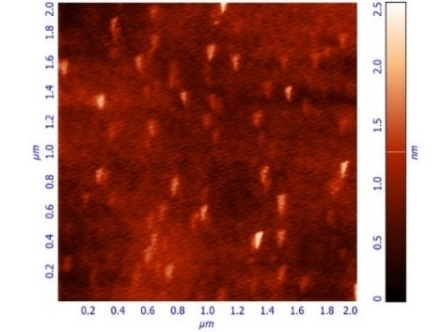 | 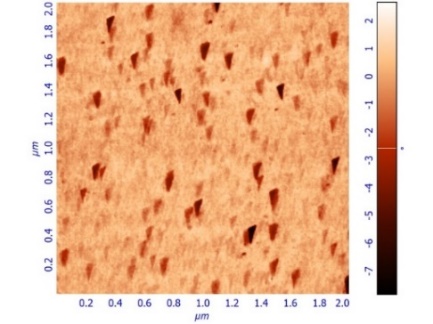 |
| ***I:SnO_2_*** | 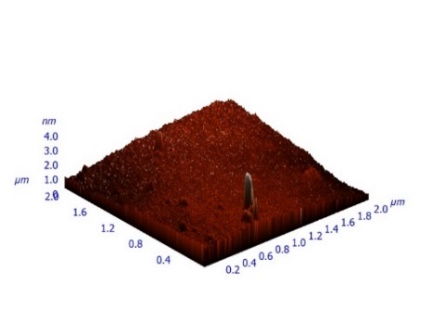 | 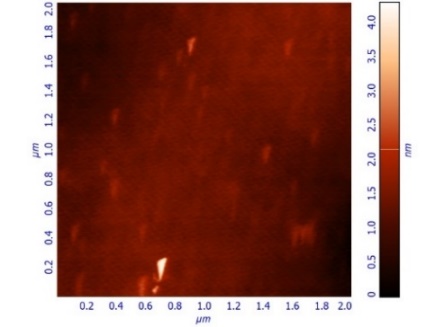 | 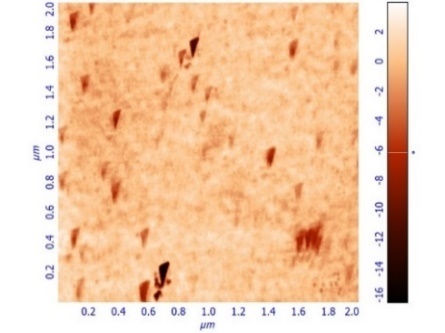 |

***Figure S6. (a)*** *The AFM images for the halogen doped samples.*

***
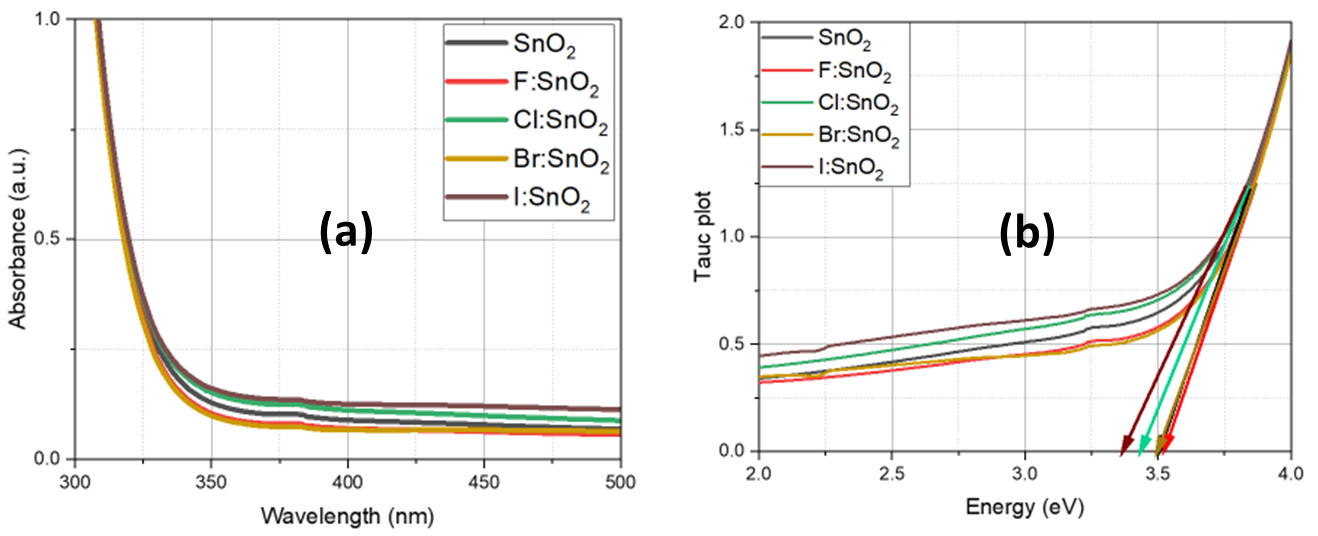
***

***Figure S7.*** ***(a)*** *UV-Vis absorption spectra and* ***(b)*** *the corresponding Tauc plots of all samples.*


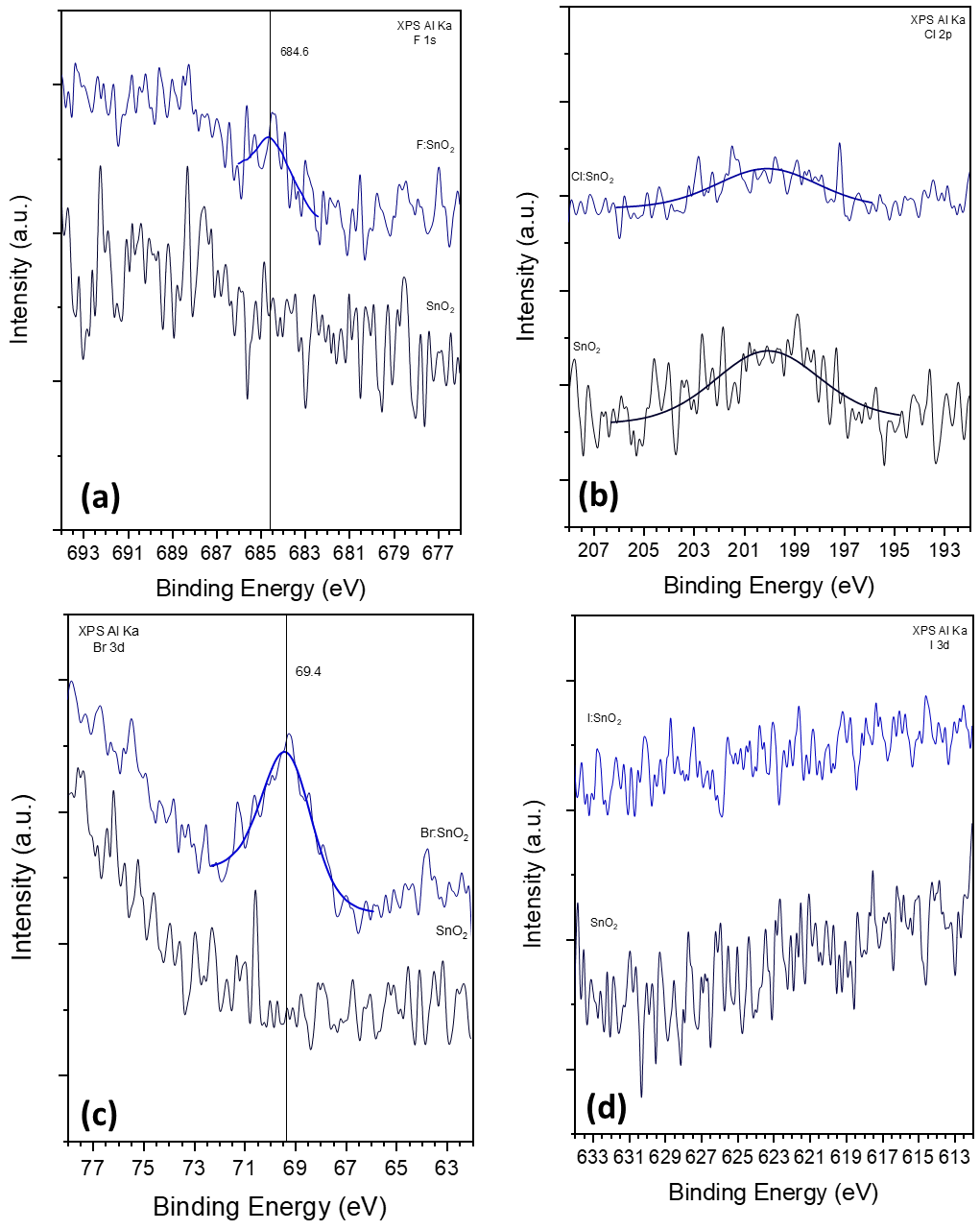


***Figure S8*** *The XP spectra for the halogen doped samples* ***(a)****F,* ***(b)****Cl,* ***(c)*** *Br and* ***(d)****I.*

***
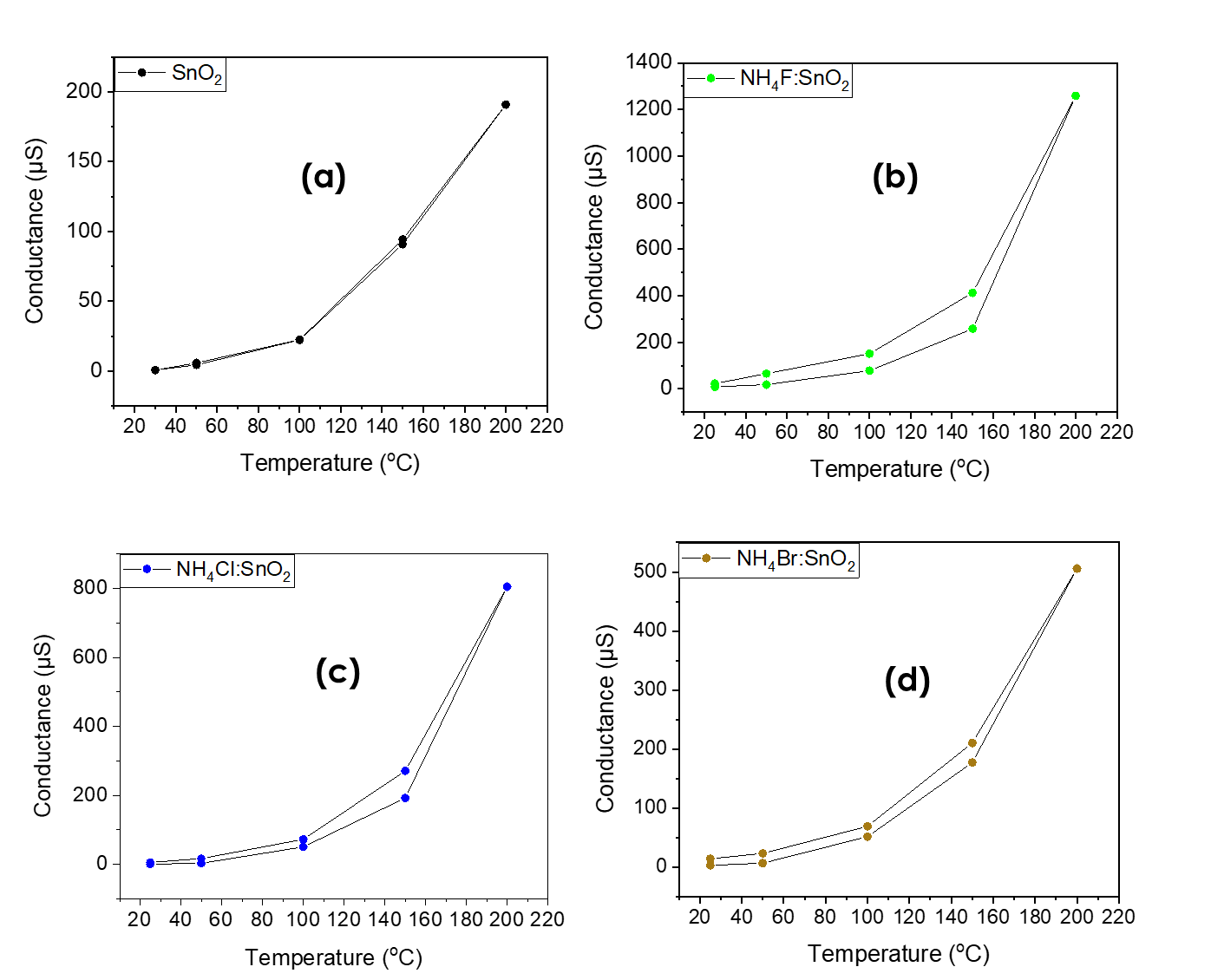
***

***
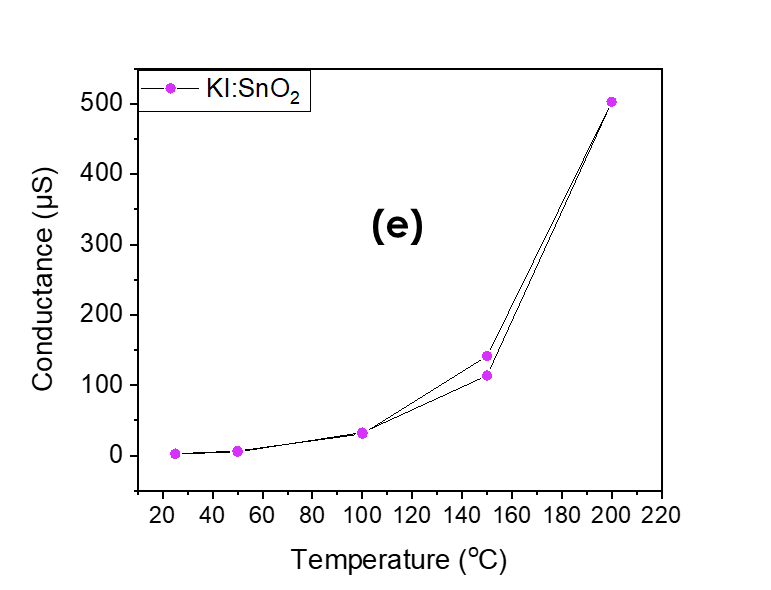
***

***Figure S9*** *The thermal cycle in dry air for* ***(a)*** *pristine SnO_2_,* ***(b)*** *F-doped,* ***(c)*** *Cl-doped,* ***(d)*** *Br-doped and (e) I-doped SnO_2_.*

(1) Segall, M. D.; Lindan, P. J. D.; Probert, M. J.; Pickard, C. J.; Hasnip, P. J.; Clark, S. J.; Payne, M. C. First-Principles Simulation: Ideas, Illustrations and the CASTEP Code. *J. Phys.: Condens. Matter* **2002**, *14* (11), 2717–2744. https://doi.org/10.1088/0953-8984/14/11/301.

(2) Perdew, J. P.; Levy, M. Physical Content of the Exact Kohn-Sham Orbital Energies: Band Gaps and Derivative Discontinuities. *Phys. Rev. Lett.* **1983**, *51* (20), 1884–1887. https://doi.org/10.1103/PhysRevLett.51.1884.

(3) Paier, J.; Marsman, M.; Hummer, K.; Kresse, G.; Gerber, I. C.; Ángyán, J. G. Screened Hybrid Density Functionals Applied to Solids. *The Journal of Chemical Physics* **2006**, *124* (15), 154709. https://doi.org/10.1063/1.2187006.
